# Author Correction: SARS-CoV-2 antibody dynamics and transmission from community-wide serological testing in the Italian municipality of Vo’

**DOI:** 10.1038/s41467-021-25321-z

**Published:** 2021-08-12

**Authors:** Ilaria Dorigatti, Enrico Lavezzo, Laura Manuto, Constanze Ciavarella, Monia Pacenti, Caterina Boldrin, Margherita Cattai, Francesca Saluzzo, Elisa Franchin, Claudia Del Vecchio, Federico Caldart, Gioele Castelli, Michele Nicoletti, Eleonora Nieddu, Elisa Salvadoretti, Beatrice Labella, Ludovico Fava, Simone Guglielmo, Mariateresa Fascina, Marco Grazioli, Gualtiero Alvisi, Maria Cristina Vanuzzo, Tiziano Zupo, Reginetta Calandrin, Vittoria Lisi, Lucia Rossi, Ignazio Castagliuolo, Stefano Merigliano, H. Juliette T. Unwin, Mario Plebani, Andrea Padoan, Alessandra R. Brazzale, Stefano Toppo, Neil M. Ferguson, Christl A. Donnelly, Andrea Crisanti

**Affiliations:** 1 MRC Centre for Global Infectious Disease Analysis and the Abdul Latif Jameel Institute for Disease and Emergency Analytics, School of Public Health, Imperial College London, London, UK; 2 Department of Molecular Medicine, University of Padova, Padova, Italy; 3 Azienda Ospedale Padova, Padova, Italy; 4 Department of Surgery, Oncology and Gastroenterology, University of Padova, Padova, Italy; 5 Department of Medicine, University of Padova, Padova, Italy; 6 Department of Statistical Sciences, University of Padova, Padova, Italy; 7 CRIBI Biotech Centre, University of Padova, Padova, Italy; 8 Department of Statistics, University of Oxford, Oxford, UK; 9 Department of Life Science Imperial College London, Exhibition Road, London, UK

**Keywords:** Computational models, Viral infection, SARS-CoV-2, Epidemiology

Correction to: *Nature Communications* 10.1038/s41467-021-24622-7, published online 19 July 2021.

The original version of this Article contained an error in the author affiliations.

Affiliation 1 incorrectly read ‘MRC Centre for Global Infectious Disease Analysis and the Abdul Latif Jameel Institute for Disease and Emergency Analytics (J-IDEA), School of Public Health, Imperial College London, London, UK’ instead of the correct ‘MRC Centre for Global Infectious Disease Analysis and the Abdul Latif Jameel Institute for Disease and Emergency Analytics, School of Public Health, Imperial College London, London, UK’.

The original version of this Article also contained an error in the Acknowledgements:

‘We acknowledge the Abdul Latif Jameel Institute for Disease and Emergency Analytics, funded by the Abdul Latif Jameel Foundation’ should have read ‘We acknowledge the Abdul Latif Jameel Institute for Disease and Emergency Analytics, funded by Community Jameel’.

These errors have been corrected in both the PDF and HTML versions of the Article.

